# Quorum Sensing Inhibitory Activity of Giganteone A from *Myristica cinnamomea* King against *Escherichia coli* Biosensors

**DOI:** 10.3390/molecules21030391

**Published:** 2016-03-21

**Authors:** Yasodha Sivasothy, Thiba Krishnan, Kok-Gan Chan, Siti Mariam Abdul Wahab, Muhamad Aqmal Othman, Marc Litaudon, Khalijah Awang

**Affiliations:** 1 Department of Chemistry, Faculty of Science, University of Malaya, Kuala Lumpur 50603, Malaysia; yasodhasivasothy@gmail.com (Y.S.); maryamabdwahab@gmail.com (S.M.A.W.); qmalxez44@gmail.com (M.A.O.); 2 Division of Genetics and Molecular Biology, Institute of Biological Sciences, Faculty of Science, University of Malaya, Kuala Lumpur 50603, Malaysia; thibavengdes@yahoo.com (T.K.); kokgan@um.edu.my (K.-G.C.); 3 Institut de Chimie des Substances Naturelles, Centre National de la Recherche Scientifique, 91198 Gif-sur-Yvette, Cedex, France; marc.LITAUDON@cnrs.fr

**Keywords:** *Myristica cinnamomea* King, Myristicaceae, acylphenols, dimeric acylphenols, anti-quorum sensing activity

## Abstract

Malabaricones A–C (**1**–**3**) and giganteone A (**4**) were isolated from the bark of *Myristica* *cinnamomea* King. Their structures were elucidated and characterized by means of NMR and MS spectral analyses. These isolates were evaluated for their anti-quorum sensing activity using quorum sensing biosensors, namely *Escherichia coli* [pSB401] and *Escherichia coli* [pSB1075], whereby the potential of giganteone A (**4**) as a suitable anti-quorum sensing agent was demonstrated.

## 1. Introduction

The increasing incidence of multi-drug resistant bacteria has prompted the search for potent, novel antibacterial drugs or complementary agents against resistant pathogens with new targets or novel mechanisms [1]. Quorum sensing is one such target mechanism. It is a cell-cell communication system used by most Gram-negative bacteria [2]. In quorum sensing (QS), bacteria use chemical signaling molecules commonly known as auto-inducers to track changes in cell population density. By monitoring the changes in the concentration of the auto-inducers, QS regulates gene expression especially virulence factor production in pathogenic bacteria [3]. Therefore, the disruption of QS is considered as an alternative for antibiotic treatment. New QS inhibitory compounds are known to constitute a new group of antimicrobial agents with applications in many fields such as medicine and agriculture [4,5].

Plants have been used for centuries in traditional medicine due to their diverse secondary metabolites. Plants grow in environments with high bacterial densities and have developed an evolutionary co-existence with QS inhibitory compounds or QS mimic compounds which reduce the pathogenic capability of bacteria [1,2]. Due to their diverse chemical repertoire, the anti-virulence properties of medicinal plants and their constituents are attracting attention since plants are able to interfere with bacterial communication process thereby disrupting associated cellular mechanisms of functions [1,2].

*Myristica cinnamomea* King (Myristicaceae), commonly known as cinnamon nutmeg, is distributed in the Malayan Peninsula, Singapore, Borneo and Philippines. Locally, it is referred to as “pala bukit” [6]. *M. cinnamomea* is a tree 15 m in height and 45 cm in diameter. Its outer bark is dark brown, rugose with fine grid cracks, while the inner bark is pale brown. The leaves are oblong to oblanceolate, bright green above and pale silvery brown below. The fruit is yellow and globose to broadly globular-oblong. Its seeds are red and used as spices [6]. Recently, we isolated two new α-glucosidase inhibitors from the hexane extract of the bark; giganteone D (IC_50_ 5.05 µM) and cinnamomeone A (IC_50_ 358.80 µM) [7]. In a previous study of ours, we identified malabaricone C isolated from the methanol extract of the bark to possess anti-QS activity against *Pseudomonas aeruginosa* PAO1 [8]. In the current work however, we decided to increase the amount of plant material and reinvestigate its chemical constituents in search of new acylphenols and dimeric acylphenols with anti-QS properties against *Escherichia coli* biosensors.

## 2. Results and Discussion

**Figure 1:**
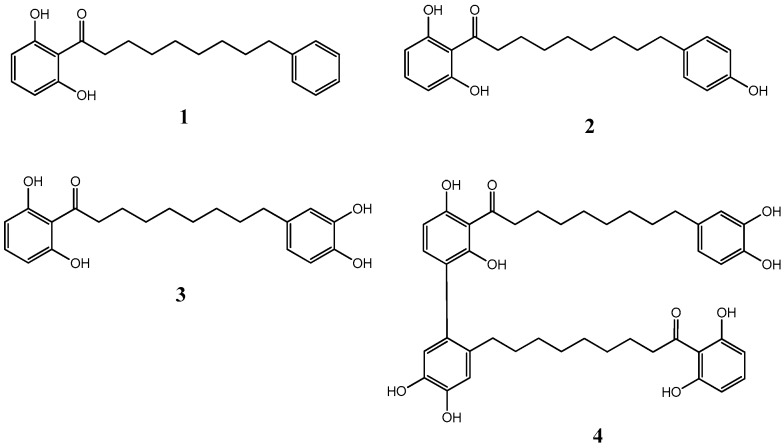
Structures of compounds **1**–**4**.

**Figure 2:**
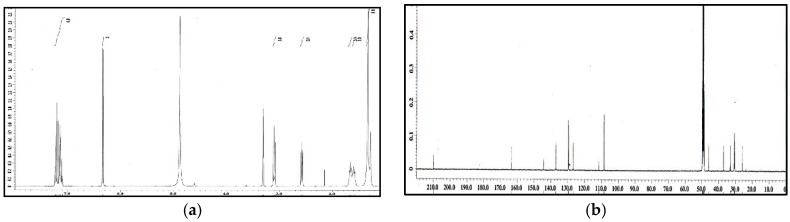
^1^H-NMR (**a**) and ^13^C-NMR (**b**) spectra of compound **1**.

**Figure 3:**
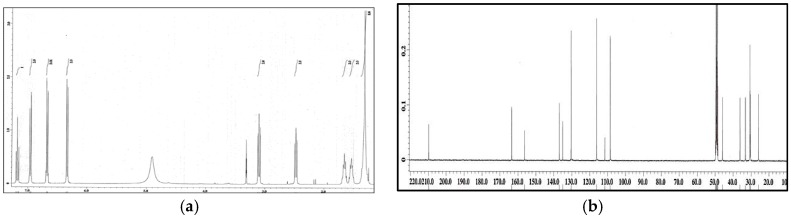
^1^H-NMR (**a**) and ^13^C-NMR (**b**) spectra of compound **2**.

**Figure 4:**
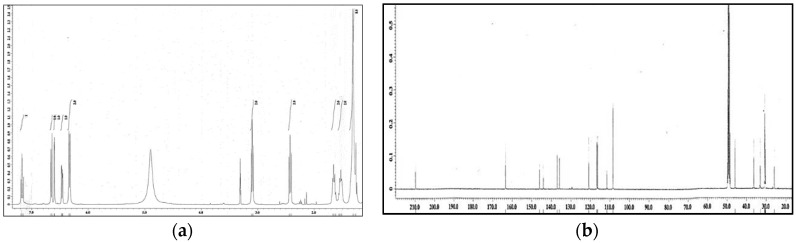
^1^H-NMR (**a**) and ^13^C-NMR (**b**) spectra of compound **3**.

**Figure 5:**
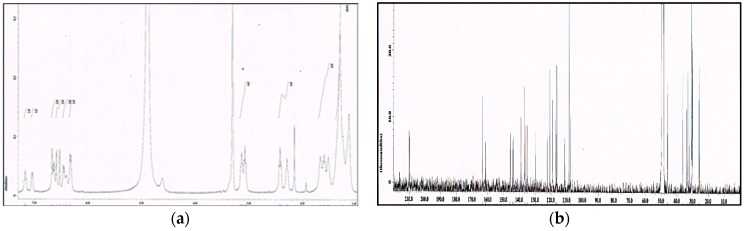
^1^H-NMR (**a**) and ^13^C-NMR (**b**) spectra of compound **4**.

The ethyl acetate soluble fraction of the acetone extract of the dried bark of *M. cinnamomea* was subjected to repeated silica gel column chromatography to yield four known acylphenols and dimeric acylphenols **1**–**4**. They were identified as malabaricone A (**1**), malabaricone B (**2**), malabaricone C (**3**) and giganteone A (**4**) (Figure 1) upon comparison of their spectroscopic data with those reported in the literature [9,10] and were further assessed for their anti-QS activity against *E. coli* QS biosensors. The ^1^H-NMR and ^13^C-NMR spectra of compounds **1**–**4** are depicted in Figure 2, Figure 3, Figure 4 and Figure 5.

**Figure 6:**
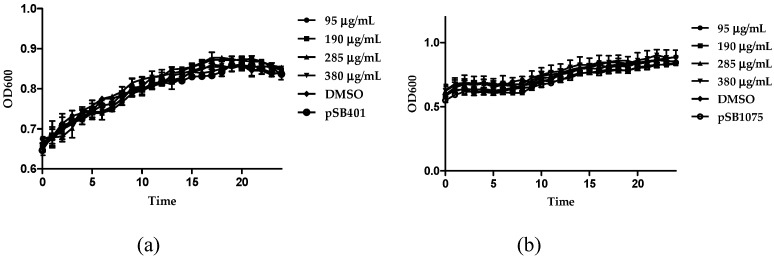
Growth effect of giganteone A with increasing concentration from 95 µg/mL (circle), 190 µg/mL (square), 285 µg/mL (triangle) to 380 µg/mL (inverted triangle) while DMSO (diamond) on (**a**) *E. coli* [pSB401] and (**b**) *E. coli* [pSB1075], respectively served as control (circle with hole). Data were analyzed by one-way analysis of variance with *p* < 0.05 being significant.

In the present study, compounds **1**–**4** were tested for possible anti-QS properties using *E. coli* [pSB401] and *E. coli* [pSB1075] as QS biosensors. These biosensor strains respond to the QS signaling molecules *N*-acylhomoserine lactones by producing bioluminescence preferentially to the presence of exogenous AHLs from six to eight carbons in length (for strain *E. coli* [pSB401]) or AHLs with acyl chains of 10–14 carbons in length (for strain *E. coli* [pSB1075]). Therefore, the reduction in bioluminescence compared to the control showed the presence of anti-QS effect. As a prerequisite, we have verified that the compounds did not show bactericidal effect on all biosensor cells (Figure 6a,b).

**Figure 7:**
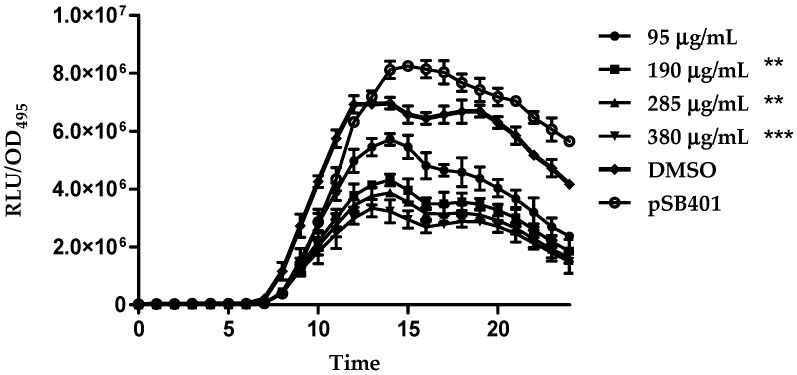
Bioluminescence expression of *E. coli* [pSB401] by giganteone A with increasing concentration from 95 µg/mL (circle), 190 µg/mL (square), 285 µg/mL (triangle) to 380 µg/mL (inverted triangle) while DMSO (diamond) and *E. coli* [pSB401] supplemented with C6-HSL, respectively served as control (circle with hole) was included. The data were presented as RLU/OD to account for any differences in growth. Data were analyzed by one-way analysis of variance with *p* < 0.05 being significant. ”**” means the value is very significant while “***” means the value is extremely significant.

**Figure 8:**
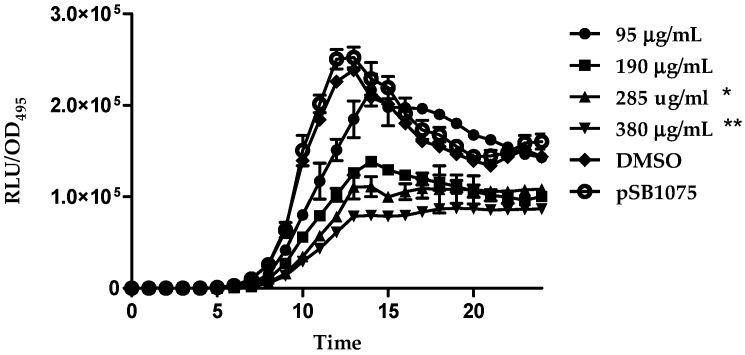
Bioluminescence expression of *E. coli* [pSB1075] by giganteone A with increasing concentration 95 µg/mL (circle), 190 µg/mL (square), 285 µg/mL (triangle) to 380 µg/mL (inverted triangle) while DMSO (diamond) as control were included. The data were presented as RLU/OD to account for any differences in growth. Data were analyzed by one-way analysis of variance with *p* < 0.05 being significant. “*” means the value is significant while ”**” means the value is very significant.

Under the present experimental conditions, increasing concentrations of compound **4** showed significant inhibition of the bioluminescence produced by both *E. coli* [pSB401] (from increasing the concentration of 95 µg/mL to 380 µg/mL) and *E. coli* [pSB1075] (from increasing concentration of 285 µg/mL to 380 µg/mL) (Figure 7 and Figure 8). DMSO (solvent) did not show any antimicrobial effects in the performed bioassays when applied at different concentrations. However, malabaricone A (**1**), malabaricone B (**2**) and malabaricone C (**3**) showed no significant bioluminescence inhibition. Thus, the current study indicates the promising anti-QS activity for both short and long chain AHL QS systems. A similar study has shown that compound **3** isolated from the methanol extract of the bark of *M. cinnamomea* also exhibited anti-QS activity [8]. Besides that, other compounds including *trans*-cinnamaldehyde [11], polyhydroxyanthraquinones [12] and furanones [13] have been reported as QS inhibitors in recent studies.

## 3. Materials and Methods

### 3.1. General Procedures

Analytical TLC was carried out on 60 F_254_ silica gel plates (absorbent thickness: 0.25 mm, Merck, Darrmstadt, Germany). Column chromatography was performed using silica gel (Merck 230–400 mesh, ASTM). IR spectra were recorded using a Perkin-Elmer Spectrum 400 FT-IR Spectrometer (Perkin Elmer, Waltham, MA, USA). NMR spectra were acquired in CD_3_OD (Merck) using a JEOL ECA 400 MHz NMR spectrometer (JEOL, Tokyo, Japan). The LCMS-IT-TOF spectra were obtained on a UFLC Shimadzu Liquid Chromatograph with a SPD-M20A diode array detector coupled to an IT-TOF mass spectrometer (Shimadzu, Kyoto, Japan). UV spectra were recorded using a Shimadzu 1650 PC UV-Vis Spectrophotometer (Shimadzu). All solvents were of analytical grade and were distilled prior to use.

### 3.2. Plant Material

*M. cinnamomea* was collected from Johor in 2003. The plant was identified by Mr. Teo Leong Eng and its voucher specimen (KL 5043) has been deposited with the University of Malaya herbarium.

### 3.3. Extraction and Isolation

Dried powdered bark (2.0 kg) of *M. cinnamomea* was extracted twice with hexane (10.0 L,) followed by acetone (15.0 L) at room temperature, affording 8.40 g and 242.56 g of extracts, respectively. The acetone extract was re-extracted twice with ethyl acetate (2.0 L) at room temperature to yield 58 g of extract. 26 g of the ethyl acetate extract was chromatographed over a silica gel column (650 g, 7.2 cm × 63 cm) eluting with dichloromethane gradually enriched with ethyl acetate (0%–100%) to provide 12 main fractions (MC 1 to MC 12). Fraction MC 2 (8.50 g; eluted with dichloromethane:ethyl acetate [80:20 *v*/*v*]) was further purified via silica gel column chromatography (210 g, 4.0 cm × 50 cm) with dichloromethane:ethyl acetate (95:5 *v*/*v*, 0.5 L) as the eluent which led to the isolation of **1** (4.80 g) and **2** (1.54 g). Fractions MC 3 and MC 4 (9.3 g, which eluted with dichloromethane:ethyl acetate [80:20 *v*/*v*]) were combined and re-chromatographed over silica gel (230 g, 4.0 cm × 50 cm) with 1.5 L of the same solvent system to afford **3** (3.43 g). Column chromatography (25 g, 2.5 cm × 30 cm) of fraction MC 5 (0.93 g; eluted with dichloromethane:ethyl acetate [60:40 *v*/*v*]) using an isocratic solvent system of dichloromethane:ethyl acetate (75:25 *v*/*v*, 0.5 L) provided sub-fractions MC 5-1 (0.01 g), MC 5-2 (0.10 g) and MC 5-3 (0.55 g). Final purification to yield **4** (127.8 mg) was achieved via column chromatography (25 g, 2.5 cm × 30 cm) of sub-fraction MC 5-3 with dichloromethane:acetone (80:20 *v*/*v*, 0.4 L) as the eluent. The purified compounds; malabaricone A (**1**), malabaricone B (**2**), malabaricone C (**3**) and giganteone A (**4**), were dissolved in 20% *v*/*v* DMSO and stored at −20 °C prior to use.

### 3.4. Biosensors and Growth Conditions

Biosensors used in this study are listed in Table 1. The strains were routinely cultured at 37 °C in Luria Bertani (LB) broth (1% *w*/*v* peptone, 0.5% *w*/*v* yeast extract, 0.5% *w*/*v* NaCl, per 100 mL distilled water) with shaking (220 rpm) and supplemented with tetracycline (20 μg/mL) [14].

### 3.5. Quantification of Bioluminescence for Anti-QS Assay

Bioluminescence production was quantified using a Tecan Infinite M200Pro microplate reader (Tecan Group Ltd., Mannedorf, Switzerland). Briefly, an overnight culture of *E. coli* biosensor cells was diluted using LB Broth to an OD_600_ of 0.1. Next, 0.2 mL of *E. coli* biosensor cells and giganteone A were added into the well of a Greiner 96-well microtitre plate with increasing concentrations from 95 µg/mL to 380 µg/mL. This step was repeated for all of the other compounds. For *E. coli* [pSB401] and *E. coli* [pSB1075], *N*-hexanoyl-L-homoserine lactone (C6-HSL, 0.2 μg/mL) and *N*-(3-oxo-dodecanoyl)-l-homoserine lactone (3-oxo-C12-HSL, 0.2 μg/mL) were supplemented, respectively. The bioluminescence and OD_495_ were determined every 30 min for 24 h at 37 °C by the microplate reader. The production of bioluminescence is given as the relative light units (RLU) per unit of optical density at 495 nm, which accounted for the influence of increased growth on the total bioluminescence [16]. Reduction of bioluminescence in *E. coli* [pSB401] and *E. coli* [pSB1075] suggested anti-QS properties. Biosensor cells treated with DMSO alone were used as the negative control. Experiments were performed in triplicates and repeated three times [17,18].

### 3.6. Statistical Analysis

All results represent the average of three independent experiments. The data were presented as mean ± standard deviation (SD) and analyzed by one-way analysis of variance (ANOVA) and Student’s *t*-test. The *p* < 0.05 was considered as significant, calculated using the GraphPad PrismVersion 5 (GraphPad Software Inc., San Diego, CA, USA).

## 4. Conclusions 

In summary, giganteone A (**4**) can be considered a QS inhibitor against *E. coli* biosensors.

## Figures and Tables

**Table 1:** List of biosensors used.

Biosensors	Description	Source
*Escherichia coli* [pSB401]	*luxRluxl*’ (*Photobacterium fischeri* [ATCC 7744]): *luxCDABE* (*Photorhabdus luminescens* [ATCC 29999]) fusion; pACYC184-derived, TetR, AHL biosensor producing bioluminescence in respond to short chain AHL	[15]
*Escherichia coli* [pSB1075]	*lasRlasl*’ (*P. aeruginosa* PAO1)*: luxCDABE* (*P. luminescens* [ATCC 29999]) fusion in pUC18 AmpR, AHL biosensor producing bioluminescence in respond to long chain AHL	[15]
